# Cellular targets for neuropeptide Y-mediated control of adult neurogenesis

**DOI:** 10.3389/fncel.2015.00085

**Published:** 2015-03-16

**Authors:** Maria Concetta Geloso, Valentina Corvino, Valentina Di Maria, Elisa Marchese, Fabrizio Michetti

**Affiliations:** Institute of Anatomy and Cell Biology, Università Cattolica del Sacro CuoreRome, Italy

**Keywords:** neuropeptide Y, neurogenesis, neurogenic niche, neural stem cells, microglia, astrocyte, endothelium

## Abstract

Neuropeptides are emerging as key regulators of stem cell niche activities in health and disease, both inside and outside the central nervous system (CNS). Among them, neuropeptide Y (NPY), one of the most abundant neuropeptides both in the nervous system and in non-neural districts, has become the focus of much attention for its involvement in a wide range of physiological and pathological conditions, including the modulation of different stem cell activities. In particular, a pro-neurogenic role of NPY has been evidenced in the neurogenic niche, where a direct effect on neural progenitors has been demonstrated, while different cellular types, including astrocytes, microglia and endothelial cells, also appear to be responsive to the peptide. The marked modulation of the NPY system during several pathological conditions that affect neurogenesis, including stress, seizures and neurodegeneration, further highlights the relevance of this peptide in the regulation of adult neurogenesis. In view of the considerable interest in understanding the mechanisms controlling neural cell fate, this review aims to summarize and discuss current data on NPY signaling in the different cellular components of the neurogenic niche in order to elucidate the complexity of the mechanisms underlying the modulatory properties of this peptide.

## Introduction

In adult tissues, stem cells reside in a permissive and specialized microenvironment, or niche, in which different molecular signals coming from the external environment, together with feedback signals from progeny to parent cells, tightly regulate self-renewal, multipotency and stem cell fate (for review see Hsu and Fuchs, 2012). In this regard, many findings underlie the key role played by neurotransmitters on stem cell biology in niches located both inside and outside the central nervous system (CNS; for review see Katayama et al., 2006; Riquelme et al., 2008). Cross-species comparative analysis points out that it could be included in a more general and evolutionary old function, going beyond their role in inter-neuronal communication (for review see Berg et al., 2013). Among them, neuropeptides, molecules released both by neurons, as co-transmitters, and by many additional release sites (for review see van den Pol, 2012), are emerging as important mediators for signaling in both neurogenic and non-neurogenic stem cell niches (for review see Oomen et al., 2000; Louridas et al., 2009; Zaben and Gray, 2013), thus representing possible shared signaling molecules in their biological dynamics.

One of the most abundant neuropeptides in the CNS is neuropeptide Y (NPY), a 36-amino-acid polypeptide that is highly conserved during phylogenesis (Larhammar et al., 1993). Through its ability to modify its levels and expression pattern following environmental changes in both physiological and pathological conditions (Scharfman and Gray, 2006; Zhang et al., 2014), it is involved in many different functions, both inside and outside the CNS. These functions are performed by binding to different G-coupled NPY receptors distributed in different organs (Pedrazzini et al., 2003).

In peripheral organs, NPY can be found in sympathetic nerves, where its release mediates vasoconstrictive effects, in adrenal medulla and in platelets (for review see Hirsch and Zukowska, 2012). NPY takes part in cardiovascular and metabolic response to stress (for review see Hirsch and Zukowska, 2012), in coronary heart disease and hypertension (Zukowska-Grojec et al., 1993). More recently, the NPY-induced modulation of different stem cell niches has been highlighted. A direct role in adipogenesis has been indicated (Kuo et al., 2007; Park et al., 2014; Zhang et al., 2014), as well as its angiogenic properties, which have been widely described in different tissues (Ekstrand et al., 2003; Zukowska et al., 2003). The NPY system is also crucially involved in the regulation of the osteogenic niche, where its presence is due to both local production and release from NPY-immunoreactive fibers, and it plays a pivotal function in the neuro-osteogenic network that regulates bone homeostasis (Franquinho et al., 2010; Lee et al., 2010, 2011).

Within the CNS, NPY is a major regulator of food consumption and energy homeostasis (for review see Lin et al., 2004), acts as one of the crucial players of the stress-related mechanisms (for review see Hirsch and Zukowska, 2012), and participates in anxiety, memory processing and cognition (for review see Decressac and Barker, 2012). It is also involved in the pathogenesis of several neurologic diseases, including neurodegenerative diseases, such as Alzheimer’s disease, Huntington’s disease (revised by Decressac and Barker, 2012) and temporal lobe epilepsy (Marksteiner et al., 1989, 1990; Vezzani and Sperk, 2004), in which anticonvulsant and neuroprotective effects have also been observed (for reviews see Vezzani et al., 1999; Vezzani and Sperk, 2004; Gray, 2008; Decressac and Barker, 2012; Malva et al., 2012). At the cellular level, it is either co-released locally by GABAergic interneurons (for review see Sperk et al., 2007; Karagiannis et al., 2009) or comes from the blood by diffusion across the blood-brain barrier (Kastin and Akerstrom, 1999). It modulates excitatory neurotransmission and regulates hyperexcitability, particularly in the hippocampus (Baraban et al., 1997). The Y1, Y2 and Y5 receptors (Y1R, Y2R, Y5R) exhibit specific distribution patterns within the CNS (Parker and Herzog, 1999; Xapelli et al., 2006) and mediate the wide range of NPY physiological functions (Pedrazzini et al., 2003).

Due to the involvement of the NPY system in many of the numerous physiological (e.g., physical activity and learning), and/or pathological stimuli (e.g., stress, seizures, neurodegenerative diseases) (Redrobe et al., 2004; Vezzani and Sperk, 2004; Decressac and Barker, 2012; Hirsch and Zukowska, 2012; Jiang et al., 2014) that strictly regulate the biological dynamics of the neurogenic niche (Kempermann et al., 2004; Zhao et al., 2008), its role in the modulation of adult neurogenesis appears particularly relevant (for review see Gray, 2008; Decressac and Barker, 2012; Malva et al., 2012; Zaben and Gray, 2013).

Interestingly, NPY-responsive cells in the CNS are known as not being confined to neurons, but they also include astrocytes (Hösli and Hösli, 1993; Barnea et al., 1998; Ramamoorthy and Whim, 2008; Santos-Carvalho et al., 2013), oligodendrocyte precursor cells (Howell et al., 2007), microglia (Ferreira et al., 2010, 2011) and endothelial cells (Zukowska-Grojec et al., 1998), which are key components of the specialized microenvironment where adult neurogenesis takes place.

In this context, a comprehensive analysis of relevant data on the NPY-mediated control of adult neurogenesis, focusing on its effects on the different cellular components of the neurogenic niche, could be particularly helpful to improve our understanding of the complex functions of this neuropeptide.

## NPY and Neural Stem Cells (NSCs)

**Figure 1:**
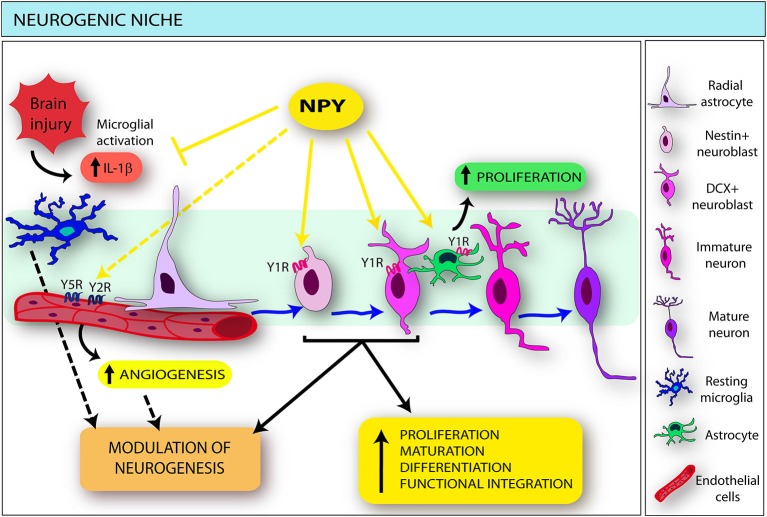
**Schematic drawing indicating the main effects exerted by neuropeptide Y (NPY) on the different components of the neurogenic niche**. NPY, released by different sources in both physiological and pathological conditions, directly targets selected neural stem cell (NSC) subtypes (namely nestin- and doublecortin [DCX]-positive cells), inducing proliferation, differentiation, migration and functional integration of newly-born neurons. NPY also modulates microglia functions: through the interaction with the Y1R, it inhibits microglial activation and interleukin (IL)-1beta release. The influence of NPY-microglia interactions in the modulation of neurogenesis (dotted black arrow) may be hypothesized. In addition, NPY stimulates astrocyte proliferation mainly via the Y1 receptors (Y1R). NPY also acts on the endothelium through the Y2 receptors (Y2R), in cooperation with the Y5 receptors (Y5R): consequently a direct effect on the endothelial component of the neurogenic niche could be hypothesized (dotted yellow arrow), resulting in increased angiogenesis and possible modulation of endogenous neurogenesis (dotted black arrow).

The direct effects of NPY on neural elements of the different neurogenic niches located outside (olfactory epithelium [OE] and retina) or inside the CNS (subventricular zone [SVZ], subcallosal zone [SCZ], subgranular zone [SGZ]) have been widely studied (Figure 1). The proximity to anatomical elements releasing NPY and the stem cell expression of Y1R, as also described in the adipogenic and osteogenic niches (Togari, 2002; Lundberg et al., 2007; Lee et al., 2010; Zhang et al., 2014), are common elements.

### Effects of NPY on the OE Niche

The vulnerability of olfactory sensory neurons to different environmental factors and the crucial role of the sense of smell in mammalian daily life account for neurogenesis in the OE; as the OE is accessible in living adult humans, it also offers a source of cells useful for understanding the biology of adult neurogenesis in health and disease (Mackay-Sim, 2010).

Hansel et al. provided the first evidence of a proliferative role of NPY on NSCs (namely basal cells) of the OE (Hansel et al., 2001), where the peptide is locally produced by the ensheathing cells of olfactory axon bundles and by sustentacular non-neuronal cells (Ubink et al., 1994).

Experiments performed using transgenic animals and primary olfactory cultures have shown that this effect is mediated by the Y1R (Hansel et al., 2001; Doyle et al., 2008) and involves Protein Kinase C and ERK1/2 pathways, which are ultimately involved in regulating the expression of genes involved in controlling cell proliferation and differentiation (Hansel et al., 2001). NPY release is regulated by ATP, which is constitutively expressed by the OE and preferentially released on injury, and the consequent activation of P2 purinergic receptors (Kanekar et al., 2009; Jia and Hegg, 2012). A role of NPY in the maturation and survival of olfactory receptor neurons has also been proposed (Doyle et al., 2012).

### Effects of NPY on the Retinal Niche

Many findings suggest the presence of a regenerative potential within the mammalian retina, in which Muller astrocytes, that are responsible for the homeostatic and metabolic support of retinal neurons, appear capable of proliferating and giving rise to neuronal cells in response to retinal damage (for review see Lin et al., 2014). Both NPY and NPY receptors (Y1R, Y2R and Y5R) are expressed by the different retinal cellular subpopulations, namely neurons, astrocytes, microglia and endothelial cells (Alvaro et al., 2007; Santos-Carvalho et al., 2014). Interestingly, *in vitro* experiments in Muller cell primary cultures pointed out a modulatory role of NPY on cell proliferation: at low dose it negatively affects the proliferation rate of the cells, while at high doses it increases cell proliferation through the Y1R stimulation and consequent activation of the p44/p42 MAPKs, p38 MAPK and PI3K (Milenkovic et al., 2004). The NPY-mediated proliferative effect has been confirmed in experiments on retinal primary cultures, which revealed that NPY-treatment stimulates retinal neural cell proliferation, through nitric oxide (NO)-cyclic GMP and ERK 1/2 pathways via Y1R, Y2R and Y5R (Alvaro et al., 2008).

### Effects of NPY on SGZ

Within the dentate gyrus (DG) NPY is selectively released by GABAergic interneurons located in the hilus, which innervate the granule cell layer in close proximity to the SGZ (for review see Sperk et al., 2007); a physiological role for NPY in the regulation of dentate neurogenesis can therefore be hypothesized. The pro-neurogenic role of NPY on hippocampal NSCs has been evidenced both *in vitro* (Howell et al., 2003, 2005, 2007) and *in vivo* (Decressac et al., 2011). *In vitro* evidence suggests a purely proliferative effect (Howell et al., 2007; Gray, 2008), specifically involving the Y1R, which is mediated by the intracellular NO pathway, through NO/cyclic guanosine monophosphate (cGMP)/cGMP-dependent protein kinase (Cheung et al., 2012), ultimately culminating in the activation of ERK1/2 signaling (Howell et al., 2003; Cheung et al., 2012). Interestingly, in line with the results obtained in the retinal niche (Alvaro et al., 2008), the role of NPY in the modulation of another signaling pathway driving a complex modulation of NSC activities emerges. It is well known, in fact, that NO exerts a dual influence on neurogenesis, depending on the source (for review see Carreira et al., 2012): while intracellular NO is pro-neurogenic, the extracellular form exerts a negative effect (Luo et al., 2010). In this respect the Y1R has also been proposed as a key target in the selective promotion of NO-mediated enhancement of dentate neurogenesis (Cheung et al., 2012).

Decressac et al. confirmed, by *in vivo* administration of exogenous NPY in both wild type and Y1R knock out mice, that NPY-sensitive cells are the transit amplifying progenitors expressing nestin and doublecortin (DCX), which selectively express the Y1R (Decressac et al., 2011), as also evidenced *in vitro* (Howell et al., 2003; Figure 1). A preferential differentiation of newly generated cells towards a neuronal lineage has also been reported (Decressac et al., 2011). In this regard, it is worth emphasizing the role also played by NPY in seizure-induced dentate neurogenesis. Studies on NPY−/− mice show a significant reduction in bromodeoxyuridine incorporation in the DG after kainic acid administration (Howell et al., 2007). Interestingly, the DCX-positive cells, besides being selective targets of NPY, are one of the most important neuroblast subpopulations recruited in seizure-induced neurogenesis (Jessberger et al., 2005). These findings are in line with the notion that different neural progenitor subpopulations within the niche show different sensitivity to physiological and/or pathological stimuli (Kempermann et al., 2004; Fabel and Kempermann, 2008), thus representing selective targets for potential drugs aimed at modulating endogenous neurogenesis, of which NPY appears to be a possible candidate.

**Figure 2:**
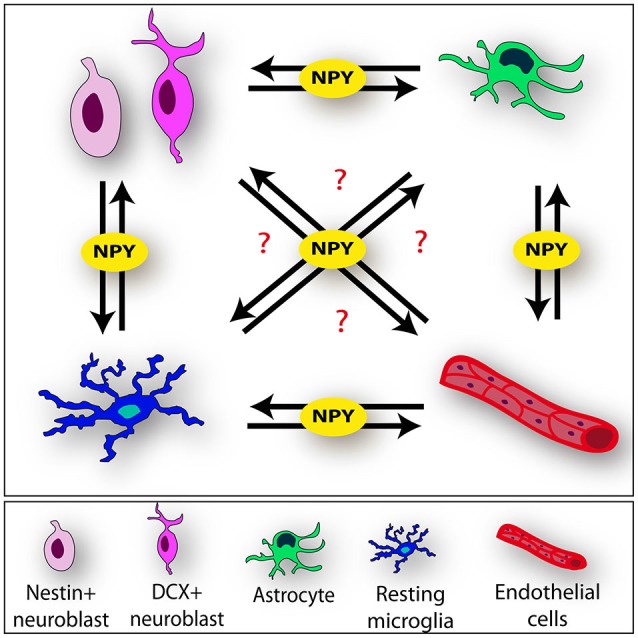
**Neuropeptide Y (NPY) mediates cell-cell interactions within the neurogenic niche**. NPY may be involved as key player of the complex communication process among the different components of the niche (neural stem cell [NSCs], microglia, astrocytes and endothelium) (black arrows).

Exogenous NPY has been administered in the Trimethyltin (TMT)-induced model of hippocampal neurodegeneration and temporal lobe epilepsy, in which selective pyramidal cell loss in hippocampal CA1/CA3 subfields (Geloso et al., 1996, 1997), reactive astrogliosis and microglial activation (for review see Geloso et al., 2011; Corvino et al., 2013; Lattanzi et al., 2013) are associated with injury-induced neurogenesis (Corvino et al., 2005). NPY injection in TMT-treated rats results in long-term effects on the hippocampal neurogenic niche, culminating in the functional integration of newly generated neurons into the local circuit (Corvino et al., 2012, 2014). The early events following NPY administration are characterized by the up-regulation of genes involved in different aspects of NSC dynamics. In particular, *Noggin*, which participates in self-renewal processes (Bonaguidi et al., 2008), *Sox-2* and *Sonic hedgehog*, both involved in the establishment and maintenance of the hippocampal niche (Favaro et al., 2009), *NeuroD1*, which regulates differentiation and maturation processes (Roybon et al., 2009), *Doublecortin*, a driver of neuroblast migration (Nishimura et al., 2014) and *brain-derived neurotrophic factor* (BDNF), which is involved in different aspects of dentate neurogenesis (Noble et al., 2011), have all been reported to be significantly modulated within the first 24 h following treatment with NPY (Corvino et al., 2012, 2014). These findings suggest that *in vivo* NPY administration, in association with the peculiar changes in the microenvironment induced by the ongoing neurodegeneration, may trigger a complex mechanism that goes beyond a mere proliferative effect. It can be speculated that it occurs as the result of NPY’s effect on both neural and non-neural elements of the niche and/or as a consequence of multiple cell-cell interactions (Figure 2).

### Effects of NPY on SVZ

In the SVZ, the most abundant reservoir of NSCs in the human brain (Doetsch, 2003b; Lim and Alvarez-Buylla, 2014), NPY comes from the cerebrospinal fluid, together with other nutrients and growth factors (Hou et al., 2006). Dense NPY-positive networks also surround this region (Stanic et al., 2008; Thiriet et al., 2011). NPY is also locally expressed by a subset of subependymal cells (Curtis et al., 2005) and by immature neural progenitors, thus suggesting a role as an autocrine/paracrine factor in the control of SVZ neurogenesis (Thiriet et al., 2011).

The effects of the peptide on the SVZ neurogenic niche have been assessed by both *in vitro* (Agasse et al., 2008; Thiriet et al., 2011) and *in vivo* studies (Stanic et al., 2008; Decressac et al., 2009). Also in this case the pro-neurogenic role of NPY is essentially played by the Y1R (Agasse et al., 2008; Stanic et al., 2008; Thiriet et al., 2011), which is mainly expressed by DCX-positive neuroblasts in adult mice (Stanic et al., 2008; Figure 1) and in Sox2 and nestin-positive cells in the developing rat (Thiriet et al., 2011). Consistently with the reported effects on dentate and olfactory NSCs, the Y1R mediates a proliferative effect, via phosphorylation of ERK MAP kinases p42 and p44 (Thiriet et al., 2011). The involvement of stress-activated protein kinase/JNK pathways, considered to play an important role in neural differentiation and maturation, has also been reported (Agasse et al., 2008).

It is well known that, while sharing common regulators, the different neurogenic niches may show some differences in specific aspects, including cellular organization, neuronal subtype differentiation and migration of NSCs (Ming and Song, 2011). In this regard, some discrepancies with the SGZ have emerged: in the SVZ, in fact, NPY appears also to exert a direct role on cell migration (Decressac et al., 2009; Thiriet et al., 2011) and neuronal differentiation (Agasse et al., 2008; Decressac et al., 2009), while a mere proliferative role, without instructive signals to differentiation processes, emerged from *in vitro* studies on SGZ NSCs (Howell et al., 2007). In particular, *in vivo* administration of NPY in adult wild type mice showed that the newly generated neurons migrate not only to the olfactory bulb, but also towards the striatum, where they preferentially differentiate into GABAergic neurons (Decressac et al., 2009). Experiments performed on Y1R knock out mice indicated that they show a disrupted assembly of neuroblasts in the rostral migratory stream, compared with the chain-like organization present in wild type animals (Stanic et al., 2008), suggesting a role of this receptor also in cell migration. The direct demonstration of a chemokinetic effect of NPY through Y1R activation and MAPK ERK1/2 pathway recruitment in NSCs, was finally given by Thiriet et al. on rat SVZ neurospheres (Thiriet et al., 2011). The possible involvement of the Y2R has also been suggested, since Y2R null mice express a reduced number of migratory neuroblasts in both the SVZ and the rostral migratory stream, with a consequently reduced number of interneurons in the olfactory bulb (Stanic et al., 2008). It should be noted, however, that the Y2R protein was found only in close proximity to rostral migratory stream associated neuroblasts, without evidence of positivity in NSCs and/or astroglial cells (Stanic et al., 2008).

Many neurodegenerative diseases induce changes in SVZ neurogenesis (Curtis et al., 2007). Alzheimer’s disease and Parkinson’s disease, for instance, are accompanied by a reduction in NSC proliferation, while stroke and Huntington’s disease cause an enhancement of SVZ neurogenesis, resulting in an increased number of new neurons, which also migrate into damaged areas (Curtis et al., 2007). Consequently, NPY administration may be of potential interest in cell replacement-based strategies for neurodegenerative diseases affecting SVZ neurogenesis. Decressac et al. demonstrated that NPY administration in the R6/2 model of Huntington disease is able to attenuate striatal atrophy and to induce a proliferative effect on SVZ NSCs (Decressac et al., 2010). However, it did not result in an increased number of newly generated neurons migrating within the striatum. NPY administration was also ineffective in modulating dentate neurogenesis in R6/2 mice. Interestingly, a reduced expression of NPY in the hilus of R6/2 mice was observed, accompanied by a reduction in the number of Y1R positive cells in the DG, thus suggesting that alterations in the NPY system might contribute to the impairment of neurogenesis in this model of Huntington disease (Decressac et al., 2010).

### Effects of NPY on SCZ

NPY also exerts its proliferative role in the SCZ, a caudal extension of the SVZ lying between the hippocampus and the corpus callosum that, in basal conditions, essentially generates oligodendrocytes migrating into the corpus callosum (Seri et al., 2006). Acting through the Y1R on nestin-positive cells (Howell et al., 2007), NPY is involved in basal and seizure-induced SCZ progenitor cell proliferation (Howell et al., 2007; Laskowski et al., 2007). Interestingly, SCZ activity appears to be modulated by seizures, resulting in the production of glial progenitors that migrate to the injured hippocampus (Parent et al., 2006), thus raising the intriguing possibility that NPY modulates SCZ oligodendrogliogenesis as well as neurogenesis (Gray, 2008).

## NPY and Microglia

Increasing evidence suggests that microglia play a relevant role in the neurogenic niche: unchallenged microglia contribute, through their phagocytic activity, to the maintenance of homeostasis of the neurogenic processes (Sierra et al., 2010), while the different functional phenotypic profiles that microglial cells undergo as a response to microenvironmental changes appear to have a dual role in neurogenesis (Carreira et al., 2012; Kettenmann et al., 2013; Su et al., 2014). Much evidence indicates how the pro-inflammatory cytokines released by activated microglia, such as interleukin (IL)-1beta, tumor necrosis factor (TNF)-alpha and IL-6, detrimentally affect neurogenesis (Ekdahl et al., 2003; Ekdahl, 2012; Su et al., 2014). On the other hand, in an enriched environment, activated microglia show proneurogenic properties via increased expression of insulin growth factor-1 (Ziv et al., 2006), while, in the presence of T-helper dependent cytokines, they reduce the production of TNF-alpha (Butovsky et al., 2006). In other words, the regulatory function of microglia in neurogenesis seems to be essentially dependent on differences in instructive signals coming from the microenvironment (Ekdahl et al., 2009).

Many studies support the modulatory role of NPY in the immune system, with effects ranging from the modulation of cell migration to macrophage and T helper cell differentiation, cytokine release, natural killer cell activity and phagocytosis, most likely through its Y1R (for review see Hirsch and Zukowska, 2012; Dimitrijević and Stanojević, 2013).

Recent findings also indicate direct interactions between NPY and microglia, the innate defensive system in the CNS (Kettenmann et al., 2013). Ferreira et al. observed that NPY, acting via the Y1R, inhibits lipopolysaccharide-induced microglial activation and reduces the associated release of IL-1beta (Ferreira et al., 2010). This effect is mediated by NPY-induced impairment of NO synthesis and reduced inducible form of nitric oxide synthase expression (Ferreira et al., 2010). In addition, NPY also induces impairment of the phagocytic properties of activated microglia (Ferreira et al., 2011) and IL-1beta-induced microglial motility (Ferreira et al., 2012). Taken together, these observations point to the key role played by the peptide in modulating the functional activities of microglia, and consequent release of mediators during inflammation (Figure 1).

Although most of these findings were obtained in *in vitro* systems, so that further research is needed in order to elucidate whether these interactions produce the same regulatory responses *in vivo*, a relevant influence of NPY-microglia interactions in the homeostasis of the neurogenic niche may be inferred. Because of the influence exerted by neuroinflammation on neurogenesis (Carreira et al., 2012), NPY-microglia signaling could be particularly relevant in the modulation of injury-induced neurogenesis. Studies exploring the interaction between neuroinflammation and neurogenesis lead to the hypothesis that the early detrimental action of microglia after acute neuronal damage can, in some situations, be modified into a supportive state during the chronic phase (Ekdahl et al., 2009) and NPY could be involved in the modulation of these transient properties of activated microglia. Many findings emphasize the ability of NSCs to modulate their own environment through the release of signaling factors (Klassen et al., 2003; Butti et al., 2014) and mutual interaction between NSCs and microglia have been shown by recent research (Mosher et al., 2012). In this regard, we may speculate that NPY, released by NSCs or coming from the surrounding environment, could be critically involved in this process, acting as a paracrine/autocrine factor which modulates both the state of activation of microglial cells and their interactions with NSCs (Figure 2).

## NPY and Astrocytes

Astrocytes are complex cells, whose supporting roles in the healthy CNS includes the regulation of blood flow, the modulation of synaptic function and plasticity and maintenance of the extracellular balance of ions and transmitters (Sofroniew, 2009). They also act as important regulators of the niche environment, through the secretion of diffusible factors (Lie et al., 2005; Barkho et al., 2006; Lu and Kipnis, 2010; Barkho and Zhao, 2011; Wilhelmsson et al., 2012) or through membrane-associated molecules (Barkho and Zhao, 2011). Thanks to their peculiar position between endothelial cells and neurons, astrocytes can mediate the exchange of molecules between vascular and neural compartments (Parpura et al., 2012). In addition, a specific subpopulation of astrocytes, the radial astrocytes, directly generates migrating neuroblasts, via rapidly dividing transit-amplifying cells (Seri et al., 2001; Doetsch, 2003a).

Several studies indicate that the expression of NPY and NPY receptors (namely Y1R) is also extended to some astrocyte subpopulations (Barnea et al., 1998, 2001; St-Pierre et al., 2000), including retinal astrocytes (Alvaro et al., 2007). It has been shown that astrocytes, like neurons, are able to synthesize NPY and show a regulated secretory pathway that is responsible for the release of multiple classes of transmitter molecules: in this regard, the activation of metabotropic glutamate receptors results in a calcium-dependent fusion of NPY-containing dense-core granules with the cell membrane and consequent peptide secretion (Ramamoorthy and Whim, 2008). It has been suggested that this process may be controlled by the RE-1–silencing transcription factor, the same factor that regulates neurosecretion in neurons (Prada et al., 2011). The expression of NPY in astrocytes is controlled by several factors: the post-natal down-regulation of glial peptide transcripts has been reported, as well as its up-regulation in adult astrocytes after brain injury (Ubink et al., 2003).

Interestingly, the *in vivo* intracerebroventricular administration of NPY significantly increases the proliferation not only of neuroblasts but also of astrocytes within the SVZ, mainly via the Y1R (Decressac et al., 2009; Figure 1). These findings delineate a complex scenario in which the peptide could exert its influence and, although direct evidence is still lacking, a role of NPY-gliotransmission in the modulation of critical steps of adult neurogenesis may be hypothesized, in both physiological and pathological conditions. In particular, it has been reported that the expression of astrocytic NPY also appears to be modulated in a cytokine-specific manner: in this regard, a relevant role of fibroblast growth factor (Barnea et al., 1998) and IL-beta (Barnea et al., 2001) in astrocytic NPY upregulation has emerged in *in vitro* studies. Both these factors can be released by astrocytes as well as by microglia: since, as previously reported, NPY inhibits microglial production of IL-1beta and IL-1beta-induced phagocytosis (Ferreira et al., 2011, 2012), a role of the peptide in astroglial/microglial interplay could be speculated. It is conceivable that it may be involved in the astrocytic regulation of microglial differentiation and activation, which, in turn, differently affect neurogenesis.

In addition, it has been reported that NPY increases the proliferative effect of the astrocyte-derived growth factor fibroblast growth factor-2 on NSCs, through the increased expression of fibroblast growth factor-receptor 1 on granule cell precursors (Rodrigo et al., 2010). This observation indicates the involvement of NPY also in the neuron-glial crosstalk and further reinforces the hypothesis that it could be one of the molecules significantly involved in the mutual interactions among the different components of the niche (Figure 2).

## NPY and the Endothelium

The vasculature is a critical component of the neurogenic niche, and endothelial cells closely interact with NSCs to form “neurovascular niches”, contributing to the regulation and maintenance of the niche (Palmer et al., 2000; Shen et al., 2004, 2008; Tavazoie et al., 2008; Goldberg and Hirschi, 2009; for review Goldman and Chen, 2011).

The molecular cross-talk between NSCs and endothelial cells is mediated by diffusible factors secreted by endothelial cells, such as BDNF and vascular endothelial growth factor (VEGF), as well as by cell-cell contact (Leventhal et al., 1999; Jin et al., 2002; Shen et al., 2004, 2008; Snapyan et al., 2009; Sun et al., 2010; for review Goldman and Chen, 2011; Vissapragada et al., 2014). Although the characterization of NPY receptors in the cerebral endothelium has not been fully clarified (Abounader et al., 1999; You et al., 2001), much evidence suggests that the endothelium could represent one of the sources, as well as one of the targets, of this peptide (Silva et al., 2005).

In this regard, different subtypes of human and rodent peripheral endothelial cells are now known to synthesize, store and constitutively express some elements of the NPY system, such as NPY itself, the Y1R and Y2R and the dipeptidyl peptidase IV, enzyme which converts NPY from the Y1R ligand to a selective agonist of Y2R (Loesch et al., 1992; Sanabria and Silva, 1994; Jackerott and Larsson, 1997; Zukowska-Grojec et al., 1998; Ghersi et al., 2001; Lee et al., 2003a; Nan et al., 2004; Silva et al., 2005; Movafagh et al., 2006; Abdel-Samad et al., 2007). NPY also acts on the endothelium, promoting angiogenesis, mainly via the Y2R, in cooperation with the Y5R (Zukowska-Grojec et al., 1998; Zukowska et al., 2003; Ekstrand et al., 2003; Lee et al., 2003a; Pons et al., 2004; Movafagh et al., 2006). VEGF- and NO-dependent pathways are primarily involved (You et al., 2001; Chen et al., 2002; Lee et al., 2003b). The hypothesis that the endothelium may represent a non-neural store of NPY, where it acts in an autocrine and in a paracrine manner, has also been proposed (Silva et al., 2005).

The angiogenic action of NPY has been confirmed in several *in vitro* and *in vivo* models: using specific receptor antagonist or transgenic Y2R knockout mice, these studies reinforced the primary role of the Y2R in mediating NPY’s angiogenic response (Zukowska-Grojec et al., 1998; Ghersi et al., 2001; Ekstrand et al., 2003; Lee et al., 2003a,b; Movafagh et al., 2006; Figure 1).

NPY also appears to exert a relevant role in the regulation and stimulation of angiogenesis in pathological processes and tissue repair, as evidenced in *in vivo* models of peripheral limb ischemia (Grant and Zukowska, 2000; Lee et al., 2003b; Tilan et al., 2013), skin wound repair (Ekstrand et al., 2003) and oxygen-induced retinopathy (Yoon et al., 2002), in which both exogenous and/or endogenous (released from neural and non-neural stores) NPY significantly contribute to tissue revascularization.

Angiogenesis and neurogenesis are related processes, as evidenced by data showing that cerebral endothelial cells activated by ischemia promote proliferation and differentiation of NSCs, while neural progenitor cells isolated from the ischemic SVZ promote angiogenesis (Teng et al., 2008). In this regard, it has also been shown that both angiogenesis and the expression of pro-angiogenic factors exert important functions in different stages of neurogenesis, such as proliferation, migration and survival (Jin et al., 2002; Louissaint et al., 2002). Interestingly, among these molecules, a relevant role is played by NO signaling, which regulates both angiogenesis and neurogenesis (Carreira et al., 2013), and whose activity is modulated by NPY not only in endothelial cells (You et al., 2001; Chen et al., 2002; Lee et al., 2003b), but also in NSCs (Cheung et al., 2012) and microglia (Ferreira et al., 2012).

It may be speculated that NPY, possibly released from the endothelium, acts as a diffusible factor that could influence and modulate elements of the neurovascular niche (Figure 2).

## Concluding Remarks and Future Perspectives

In summary, existing data provide evidence that NPY modulates the neurogenic niche performing a pro-neurogenic role directly on the NSCs, while the possibility of a concomitant modulatory action on astrocytes, microglia and endothelium activities within the niche is also possible. The involvement of NPY as a key player in the complex process of communication among the different components of the niche may be speculated, and, in this regard, there is evident need for further research to definitely elucidate the mechanisms of NPY-modulated cell/cell interactions. This could yield a more heightened understanding of some critical steps of the complex mechanisms that regulate adult neurogenesis, thus possibly providing knowledge useful to identify selective targets for potential drugs aimed at modulating NSC fate. Moreover, due to the significant involvement of the NPY system also in non-neural stem cell niches, this information could contribute to clarify the systemic role of the peptide, which appears to be involved in a set of basic homeostatic body functions, ranging from food consumption and energy homeostasis to the regulation of stem cell biology in adult tissues.

## Authors and Contributors

**MCG**: She gave substantial contributions to both the conception and design of the work; she contributed to the acquisition, analysis, and interpretation of data. She drafted the work and revised it critically. She gave the final approval of the version to be published. She agrees to be accountable for all aspects of the work in ensuring that questions related to the accuracy or integrity of any part of the work are appropriately investigated and resolved.

**VC**: She gave substantial contributions to the design of the work; she contributed to the acquisition, analysis, and interpretation of data for the work. She drafted the work and revised it critically. She gave the final approval of the version to be published. She agrees to be accountable for all aspects of the work in ensuring that questions related to the accuracy or integrity of any part of the work are appropriately investigated and resolved.

**VDM**: She contributed to the acquisition of data for the work. She drafted the work. She gave the final approval of the version to be published. She agrees to be accountable for all aspects of the work in ensuring that questions related to the accuracy or integrity of any part of the work are appropriately investigated and resolved.

**EM**: She contributed to the acquisition of data for the work. She drafted the work. She gave the final approval of the version to be published. She agrees to be accountable for all aspects of the work in ensuring that questions related to the accuracy or integrity of any part of the work are appropriately investigated and resolved.

**FM**: He provided substantial contributions to the design of the work; he contributed to the interpretation of data for the work. He revised critically the work. He gave the final approval of the version to be published. He agrees to be accountable for all aspects of the work in ensuring that questions related to the accuracy or integrity of any part of the work are appropriately investigated and resolved.

## Conflict of Interest Statement

The authors declare that the research was conducted in the absence of any commercial or financial relationships that could be construed as a potential conflict of interest.
